# A Mobile Applet for Assessing Medication Adherence and Managing Adverse Drug Reactions Among Patients With Cancer: Usability and Utility Study

**DOI:** 10.2196/50528

**Published:** 2024-02-29

**Authors:** Chenxu Ni, Yi-fu Wang, Yun-ting Zhang, Min Yuan, Qing Xu, Fu-ming Shen, Dong-Jie Li, Fang Huang

**Affiliations:** 1 Shanghai Tenth People’s Hospital Shanghai China

**Keywords:** WeChat applet, usability testing, utility testing, cancer patients, patients, cancer, qualitative study

## Abstract

**Background:**

Medication adherence and the management of adverse drug reactions (ADRs) are crucial to the efficacy of antitumor drugs. A WeChat applet, also known as a “Mini Program,” is similar to the app but has marked advantages. The development and use of a WeChat applet makes follow-up convenient for patients with cancer.

**Objective:**

This study aimed to assess the usability and utility of a newly developed WeChat applet, “DolphinCare,” among patients with cancer in Shanghai.

**Methods:**

A qualitative methodology was used to obtain an in-depth understanding of the experiences of patients with cancer when using DolphinCare from the usability and utility aspects. The development phase consisted of 2 parts: alpha and beta testing. Alpha testing combined the theory of the Fogg Behavior Model and the usability model. Alpha testing also involved testing the design of DolphinCare using a conceptual framework, which included factors that could affect medication adherence and ADRs. Beta testing was conducted using in-depth interviews. In-depth interviews allowed us to assist the patients in using DolphinCare and understand whether they liked or disliked DolphinCare and found it useful.

**Results:**

We included participants who had an eHealth Literacy Scale (eHEALS) score of ≥50%, and a total of 20 participants were interviewed consecutively. The key positive motivators described by interviewers were to be reminded to take their medications and to alleviate their ADRs. The majority of the patients were able to activate and use DolphinCare by themselves. Most patients indicated that their trigger to follow-up DolphinCare was the recommendation of their known and trusted health care professionals. All participants found that labels containing the generic names of their medication and the medication reminders were useful, including timed pop-up push notifications and text alerts. The applet presented the corresponding information collection forms of ADRs to the patient to fill out. The web-based consultation system enables patients to consult pharmacists or physicians in time when they have doubts about medications or have ADRs. The applet had usabilities and utilities that could improve medication adherence and the management of ADRs among patients with cancer.

**Conclusions:**

This study provides preliminary evidence regarding the usability and utility of this type of WeChat applet among patients with cancer, which is expected to be promoted for managing follow-up among other patients with other chronic disease.

## Introduction

Medication adherence is defined by the World Health Organization as the extent to which a person’s medication-taking behavior corresponds with agreed recommendations from health care providers [1]. Medication adherence is crucial to the efficacy of antitumor drugs. Patients with cancer have disproportionately higher burdens of comorbid chronic conditions compared to individuals without a cancer history [2]. For individuals with preexisting chronic conditions, a new cancer diagnosis can lead to tremendous challenges, including the coordination of carers and dependents as well as the management of multiple medications for comorbid conditions alongside cancer treatment [3]. Antitumor drugs are known for their numerous adverse drug reactions (ADRs), which can diminish adherence to treatment and cause medical complications. Several interventions have been developed to improve medication adherence and manage ADRs in patients with cancer [4-6]. However, the effectiveness of these interventions is controversial.

WeChat is a type of social networking software that provides flash messaging services on smart terminals. In 2020, the number of monthly active WeChat users exceeded 1.1 billion, rendering it the most common smartphone app in China. It is no longer a simple social platform but has penetrated into all aspects of people’s lives, including their health [7]. With the constant development of WeChat tools, a new development environment and platform was built for the WeChat applet used by 400 million Chinese users every day [8].

The WeChat applet, also known as the “Mini Program,” is similar to the app but has marked advantages. The steps for using the WeChat applet have been simplified, and the applet can be opened directly without downloading the app package. Interestingly, there is an independent storage space among different WeChat applets. If one no longer uses the applet, one need only close the page without uninstalling the program or clearing the cache, which is convenient for users. In addition, the IT infrastructure of WeChat applets can bring about a rapid transfer of digital data between patients with cancer and doctors or pharmacists, and reduce their burden related to oncotherapy information through real-time communication. All of these make follow-up convenient for patients with cancer by using the WeChat applet

Norman and Skinner [9] reported that participants who had a high eHealth Literacy Scale (eHEALS) score indicated that they had higher literacy skills in using the internet as a resource to obtain health information. This study selected and interviewed dozens of patients with cancer with high eHEALS scores from a tertiary hospital in Shanghai, who are willing to use an eHealth tool—the WeChat applet. If the WeChat applet can be successfully promoted in Shanghai, it will be spread sequentially in various cities throughout China. At present, the vast majority of WeChat applets are free to use, which can reduce the economic burden of follow-up among patients with cancer. However, the existing WeChat applets were developed without involving relevant stakeholders (such as health care professionals or patients) and were not subjected to mobile health app guidelines [10,11].

Usability is described as the extent to which a product can be used by specified users to achieve specified goals with effectiveness, efficiency, and satisfaction in a specified context of use [12]. Utility is defined as the functionality of the app and how useful it is to users [12]. The development phase consisted of 2 parts: alpha and beta testing. Beta testing was conducted using in-depth interviews. Alpha testing combined the theory of the Fogg Behavior Model and the usability model. The Fogg Behavior Model [13] suggests that 3 core elements—motivation, ability, and trigger—must converge at the same time for a desired behavior to take effect. Motivation and ability can be balanced against each other; for example, patients may be willing to perform a difficult task if they are highly motivated by the promise of better health outcomes. Ability refers to the patients’ skill or dexterity with respect to the simplicity (or otherwise) of using the app. A trigger is a stimulus that prompts patients to adopt and use the app (eg, an acute attack or clinician’s suggestion).

To our knowledge, no study has assessed the usability and utility of a WeChat applet for patients with cancer once it has been developed. This study aimed to assess the usability and utility of a newly developed WeChat applet—DolphinCare—among patients with cancer in Shanghai.

## Methods

### Study Design

A qualitative methodology was used to obtain an in-depth understanding of the experiences of patients with cancer when using DolphinCare from the usability and utility aspects.

### Ethical Considerations

Ethics approval was obtained from the ethics committee of Shanghai Tenth People’s Hospital prior to the study (SHSY-IEC-5.0/22K99/PO1). The study was registered in Chinese Clinical Trial Registry (ChiCTR2200058189). Written informed consent was obtained from all study participants.

### eHEALS

The modified eHEALS was used to assess participants’ literacy skills in using their smart devices to find health-related information on the internet (Multimedia Appendix 1).

### Participants

We included participants who had an eHEALS score of ≥50% and were taking 2 or more prescribed medications for their tumor treatment and chronic conditions. We excluded participants aged <18 years or those who had mental disabilities. Purposive sampling was used to recruit older (≥65 years of age) and younger (<65 years of age) participants, as we required the experiences of older participants who may have more comorbidities but may not be comfortable using mobile apps, as well as younger participants who may have fewer comorbidities (than older patients) but may be more comfortable using mobile apps. The purpose of recruiting participants based on age was to obtain wider perspectives when using DolphinCare.

### Alpha and Beta Testing in the Development Phase

**Figure 1:**
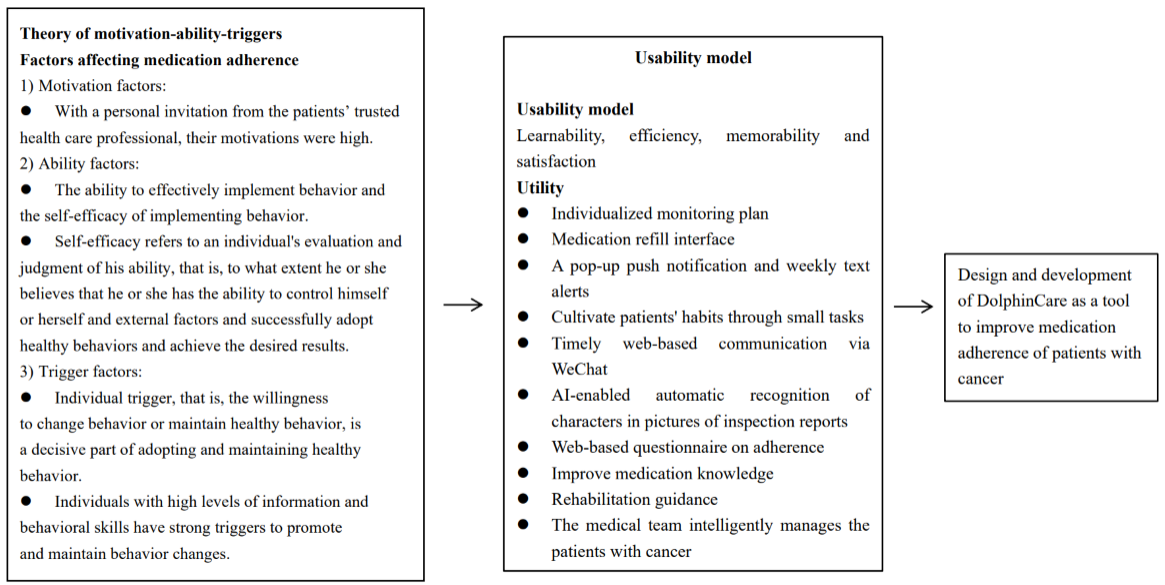
The conceptual framework for the design and development of DolphinCare based on the Fogg Behavior Model's theory of Motivation-Ability-Triggers and the usability model. AI: artificial intelligence.

The development phase consisted of 2 parts: alpha and beta testing. Alpha testing involved testing the design of DolphinCare using a conceptual framework (Figure 1), which combined the theory of the Fogg Behavior Model of Motivation-Ability-Triggers and the usability model. Our framework also included factors that could affect medication adherence and ADRs (Table 1).

**Figure 2:**
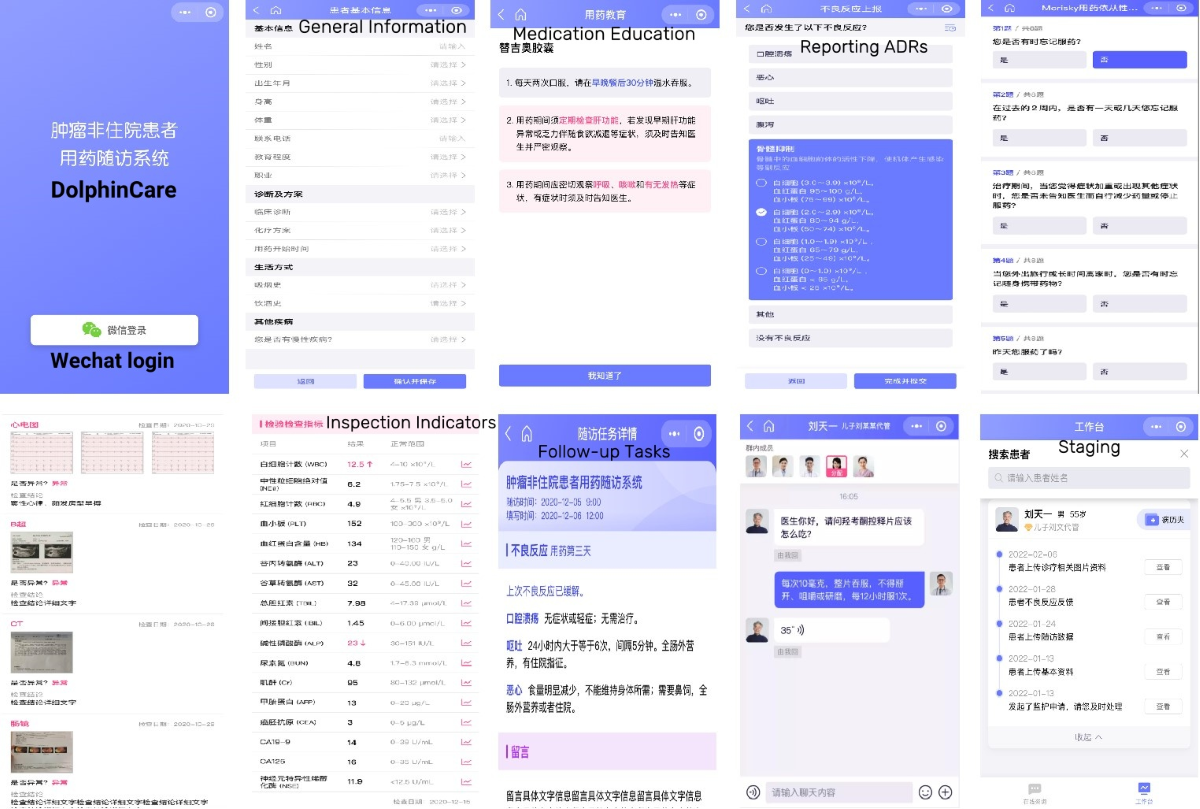
The landing page, registration page, home page, and pharmaceutical care page of DolphinCare. ADR: adverse drug reaction.

DolphinCare was then used for beta testing (Figure 2). Beta testing was conducted using in-depth interviews from July to September 2022. The first and second rounds of in-depth interviews were both conducted at the resting area of the ward. In-depth interviews were conducted to explore the views of patients with cancer regarding the usability and utility of DolphinCare when using it for the first time. We supplemented this interview process by observing the participants and documenting these observations as field notes. This allowed us to determine whether they encountered any difficulties and whether they liked or disliked the utility of DolphinCare. In-depth interviews allowed us to focus on the individual, assist the individual in using DolphinCare, and create an environment where the individual would be able to express his or her views without being influenced by others.

**Table 1:** Summary of the preferred features and utilities of DolphinCare.

Utility	Description
Individualized monitoring plan	The patients can obtain professional evaluation and guidance by implementing the established process and providing feedback to the medical team. Monitoring plan includes objectives, key indicators, medication list, follow-up plan, and precautions
Cultivate patients' habits through small tasks	After each task is completed, the page assigns the patient a “star” as a reward
A pop-up push notification and weekly text alerts	A pop-up push notification, with a “single-click” return to the app, was preset every other day. Apart from a pop-up push notification, there are weekly text alerts as well. Most patients will use the applet after receiving the notification
Complex medication regime	Ability to aid patients in managing complex medication regimens, such as a drug combination or changing a medical prescription
Timely WeChat web-based communication	In case of emergencies or questions during the treatment, patients can consult the medical team at the WeChat consultation window to solve the problems quickly and effectively
The Medical team intelligently manages the patients with cancer	The medical team can see the number of existing patients, their medication situation, adverse drug reactions, examination information, etc. They consider patients at the center for full-dimensional data monitoring and comparison. Adverse drug reactions and inspections are monitored
AI^a^-enabled automatic recognition of characters in pictures in inspection reports	AI automatically recognizes the words and data in the examination documents and inputs them into the patient database
Web-based questionnaire on adherence	Users are able to assess their adherence to medications
Rehabilitation guidance	Based on to the patient's condition and medication situation, the medical team gives knowledge guidance such as rational medication and nutritional rehabilitation

^a^AI: artificial intelligence.

### The Interview Questions

The following questions were asked to the participants during the interviews:

- What is your initial impression of using DolphinCare?
- What is your feeling about using DolphinCare after a period of use?
- What motivates you to use DolphinCare?
- Can you operate DolphinCare yourself, or do you need help from others?
- Will you continue to use DolphinCare?
- What’s the operation interface of DolphinCare that left a deep impression on you?
- What’s function do you expect DolphinCare to further improve on?
- What do you think are the inconveniences of using DolphinCare?

### Data Collection Process

Participants used their mobile WeChat app to acquire the official account of DolphinCare free of charge and then filled in the demographic form. Each participant was interviewed twice. During the interview, the “first impression” of participants in using DolphinCare would be captured. The researcher took detailed notes and observed for nonverbal cues during each interview. Facial expressions and body language subconsciously portrayed by the participants were noted down by the researchers. All interviews were audio recorded. The 8-item Morisky Medication Adherence Scale (MMAS-8) [14-16] is a questionnaire designed to facilitate the identification of barriers and behaviors associated with adherence to medication. The possible answers to questions 1 to 7 are “yes” (0 points) or “no” (1 point). Five of the questions are scored in reverse (ie, yes=1 and no=0). The possible answers to question 8 are “Never” (1 point), “Occasionally” (0.75 points), “Sometimes” (0.50 points), “Often” (0.25 points), and “All the time” (0 points) [17].

### Data Analysis

All interviews were transcribed verbatim. An interpretive-descriptive approach was used to identify the themes that emerged from the data. This approach was used to obtain a deeper understanding of the usability and utility of the perspectives and experiences of patients with cancer in using the WeChat applet. The researchers reflected on the data and began constructing an interpretive account of what the codes signified from the participants’ perspectives, and its application in clinical practice [14]. The researchers also referred to the field notes for reflections, facial cues, and body languages observed during the interviews. The research team then met to discuss the coding of the transcripts. Any coding discrepancies were resolved through discussion until a consensus was reached.

## Results

### Participants

A total of 22 participants were recruited (Table 2) for the first interview. Only 20 participants (12 men and 8 women; 16, 80% of them being patients and 4, 20% being carers) were interviewed consecutively, as 2 (P13 and P14) declined to participate. The mean age of the patients and their carers was 62.4 (SD 10.25) years. The average of the number of medications among patients was 2.7.

**Table 2:** Demographic characteristics of the recruited participants.

ID	Gender	Age (years)	Medications, n	Patient or carer	iPhone or Android user	eHEALS^a^ score (%)
P1	Male	64	3	Carer	Android	75
P2	Female	67	1	Patient	Android	56
P3	Female	73	2	Patient	iPhone	80
P4	Female	73	2	Carer	Android	78
P5	Female	35	4	Patient	Android	57
P6	Male	72	6	Patient	Android	75
P7	Male	55	3	Patient	Android	69
P8	Male	62	4	Patient	Android	69
P9	Male	47	2	Patient	iPhone	75
P10	Male	68	2	Carer	iPhone	75
P11	Male	67	3	Patient	Android	59
P12	Female	75	4	Patient	Android	57
P13	Male	65	5	Patient	Android	84
P14	Male	71	1	Patient	Android	50
P15	Male	59	1	Patient	Android	62
P16	Female	64	2	Patient	Android	71
P17	Male	50	3	Patient	Android	88
P18	Female	62	3	Patient	Android	65
P19	Male	73	4	Patient	iPhone	65
P20	Male	61	2	Carer	Android	80
P21	Female	53	2	Patient	iPhone	82
P22	Male	68	1	Patient	Android	73

^a^eHEALS: eHealth Literacy Scale.

### Adoption

We used the motivation, ability, and trigger categories of the Fogg Behavior Model to explore why patients adopted the WeChat applet using data from the qualitative interviews and usage data.

#### Adoption: Motivation

The key positive motivators described by the interviewers were to be reminded to take their medications, to alleviate their ADRs through monitoring, and to aid in medical research. Some patients had more than 1 motivation for adoption.

The motivation is to observe if I can improve the medication adherence, and manage my ADRs.

Patient 10, 68 years, male

It [taking part in this research] could be helpful for the medical studies or something. So to advance their work.

Patient 4, 73 years, female

My main motivation is to participate in this research for distracting me from my anxiety about the disease.

Patient 7, 55 years, male

#### Adoption: Abilities

Most of the patients were able to activate and use the WeChat applet by themselves. Some (typically older) patients needed assistance from younger family members.

I’m not very good with these things but somebody younger in my family would help me for handling it.

Patient 2, 67 years, female

I know how to use it … I think it is quite easy and straight forward because the layout is very clear (while showing the Homepage).

Patient 5, 35 years, female

#### Adoption: Triggers

Most patients indicated that they would receive follow-up through the WeChat applet if it was recommended by their known and trusted health care professionals.

My trusted physician recommended me to use this ‘wechat applet’ to improve my medication adherence and better manage ADRs, which makes me willing to use it.

Patient 8, 62 years, male

### Usability Testing

They were challenges encountered when adding a new medication, with regard to patients’ understanding of their complex medication regime.

#### Challenges Encountered When Adding a New Medication

Several subthemes emerged under this theme: confusion by terms used when adding medications into DolphinCare, unfamiliarity with the entered generic name of the medication, and patients’ incomprehension of their complex medication regimen.

#### Unfamiliarity With the Generic Name of the Medication

When entering medication details into the WeChat applet, most patients knew their medication by brand names but not generic names. DolphinCare requires users to enter the generic name of the medication, as the pharmacy label only contains the generic name.

I am not sure of accurately different names of my medications, and I almost always have my pill boxes with me.

Patient 15, 59 years, male

I’m not too sure what is the precise name of my medication. I only know it by its brand name. So having the indication automatically linked to the medication name is good to have.

Patient 16, 64 years, female

### Utility Testing

Two themes emerged from the utility testing of DolphinCare: utilities that could improve medication adherence and the management of ADRs.

#### A Medication Reminder System

All participants found the medication reminder useful, including timed pop-up push notifications and text alerts.

Oh yes this was helpful. It prompted me to remind my mum to take her medications.

Patient 20, 61 years, male

It would like to set timed pop-up push notifications to depend on what time I wake up in the morning and go to bed in the evening.

Patient 18, 62 years, female

#### A Medication Adherence Scoring System

Medication adherence among patients was evaluated using the MMAS-8. When a patient uses the WeChat applet for more than 1 month, a questionnaire to evaluate their medication adherence will be provided to the patient to fill in.

I’m sorry that I wasn’t aware of the MMAS-8 questionnaire until you reminded me. Happily, my medication adherence improved after using the applet.

Patient 9, 47 years, male

#### The Management of ADRs

When patients input the antitumor medication they are taking, the applet interface automatically matches the corresponding educational information of ADRs. A week later, the applet provides the corresponding collection information forms of ADRs to the patient to fill out. Health care professionals receive messages or provide web-based medication guidance to the patient based on the severity of ADRs. In addition, the WeChat applet can intelligently recognize the inspection and image reports uploaded by the patient in paper-photo versions. The WeChat applet converts the reports into text formats, and organizes and records them in the patient’s file. When there are obvious abnormalities in indicators of the reports related to ADRs, health care professionals can promptly contact the patient.

#### The Web-Based Consultation System

The web-based consultation system enables patients to consult pharmacists or physicians in time when they have doubts about medications or have ADRs. There will be a professional exclusively in charge of web-based consultation services during working days.

Oh this is good. This saved me the time and efforts to go to the hospital for consultation, and the response I got from online consultations was equally satisfactory.

Patient 17, 50 years, male

## Discussion

### Principal Findings

DolphinCare was designed and developed on the basis of the Fogg Behavior Model. This model comprised 3 phases—motivation, ability, and triggers—which suggests that a patient is able to achieve a target behavior if he or she has high motivation, ability, and an effective trigger simultaneously. The requirement phase was based on utilities that could improve medication adherence and manage ADRs, which led to the design and development of DolphinCare until the medical personnel and patients were satisfied with the prototype. This paper focused on the usability and utility testing of DolphinCare, for which patients with cancer were recruited to use the WeChat applet and provided feedback.

To our knowledge, no previous study has reported the experiences of patients with cancer when using a WeChat applet. The patients also preferred a summary page of medication, which was accessible by tapping on the medication icon on the home page of DolphinCare; this was useful as it provided a brief overview of their medication regimen. This further enhanced the usability of DolphinCare, as it would be more patient-centered and more likely to be adopted. It is worth noting that there was no particularly negative feedback provided by the participants. This is a limitation of the study, and future research needs explore the negative feedback from patients to better improve DolphinCare.

The steps to add a medication were simplified and displayed in a layered order to prevent cognitive overload [18]. However, some participants struggled when adding a new medication due to the complexity of this task. It is challenging to input data into a small device, as it requires the user to navigate the app on a small screen [19]. Despite the challenges encountered by patients, the process of adding a new medication “manually” benefited patients with regard to their medication knowledge. Participants had to “learn” the generic name of their medications, their administration frequency, and their purpose, which is beneficial for the patient's treatment process.

Several studies have shown that behavioral change is achievable through active reminders, which strengthens the benefits of medication adherence apps [20,21]. A review by Santo et al [22] in 2016 revealed that only 56% of medication adherence apps adjust flexible scheduling for medication reminders. Medication reminders with flexible scheduling (where users may opt for medication reminders on alternate days or on a weekly basis) allows for personalization of the app to suit their individual needs. Participants also reported that they had a better understanding of the frequency and indication of the medication, which appeared on the reminder, thereby improving their medication knowledge. Improved patient knowledge is known to enhance their medication adherence and clinical health outcomes [23]. However, areas involving strategies to improve patients’ medication knowledge require further investigation [24]. DolphinCare offers a pop-up push notification, with a “single-click” return to the WeChat applet, which was preset every 2 days. Besides a pop-up push notification, weekly text alerts were also provided. These allowed users who were unable to take their medications at a specific time point to take them later, which actively prompted the patients to take their medications properly and on time. DolphinCare required users to acknowledge the reminder that, theoretically, would make the patients more conscious of their adherence to medications.

Symptom monitoring is especially important for patients with cancer because they can experience varying acute and chronic side effects from their treatment regimen [25]. Egbring et al [26] conducted a 3-arm randomized controlled trial for 6 weeks with 139 patients with early-stage breast cancer undergoing chemotherapy. The participants were randomly assigned to a control group, an unsupervised group that used a mobile app to record data without physician review, or a supervised group that recorded data in the app with physician review. The results revealed that participants who had physician collaboration when using the health tracking app demonstrated increased reporting of adverse effects of chemotherapy, more precise health data entries, and stabilization of daily functional activities measured using the ECOG (Eastern Cooperative Oncology Group) scale. Our study affirmed the importance of symptom monitoring among patients with cancer, as these patients can also experience significant side effects from their treatment regimen, which influence their overall quality of life during and after their course of therapy.

The wide demographic range of the participants of this study delineates the experiences of both young and old users. The usability and utility testing of DolphinCare demonstrated the needs of patients with cancer and their caregivers better and helped tailor the WeChat applet to suit their needs. This ensured that DolphinCare would be a more patient-centered WeChat applet and more likely to be used. Our study suggests that DolphinCare can aid patients with cancer and even those with other chronic diseases to improve medication adherence and manage ADRs.

### Conclusions

DolphinCare was designed and developed on the basis of the Fogg Behavior Model. This study provides preliminary evidence of the usability and utility of this type of WeChat applet among patients with cancer. This WeChat applet had usabilities and utilities that could improve the patients’ medication adherence and ADR management.

## Appendix

Multimedia Appendix 1 eHealth Literacy Scale.

## Abbreviations

ADR: adverse drug reaction
ECOG: Eastern Cooperative Oncology Group
eHEALS: eHealth Literacy Scale
MMAS-8: 8-item Morisky Medication Adherence Scale
