# Supplementary material for: A Mobile Applet for Assessing Medication Adherence and Managing Adverse Drug Reactions Among Patients With Cancer: Usability and Utility Study
Source: JMIR Form Res. 2024 Feb 29;8:e50528. doi: 10.2196/50528 (PMC10940974; doi:10.2196/50528)
Supplement: Multimedia Appendix 1 [file formative_v8i1e50528_app1.docx]

**Appendix 1.** Participant Survey Items

A1. Have you ever looked for information about health or medical topics from any source?

 Yes

 No

A2. The most recent time you looked for information about health or medical topics, where did you go first?

*Mark only one.*

 Books

 Brochures, pamphlets, etc.

 Cancer organization

 Family

 Friend/Co-worker

 Doctor or health care provider

 Internet

 Library

 Magazines

 Newspapers

 Telephone information number

 Complementary, alternative, or traditional medicine practitioner

 Social media site, such as *weibo*

 Social media site for patient communities

 Other Specify: ______________________

Based on your most recent search for information about health, how much do you agree or disagree with the following statements?

A3. The information you found was hard for you to understand

 Strongly agree

 Somewhat agree

 Somewhat disagree

 Strongly disagree

A4. Overall, how confident are you that you could get advice or information about health or medical topics if you needed it?

 Completely confident

 Very confident

 Somewhat confident

 A little confident

 Not confident at all

A5. How often do you find numerical information easy to understand? By numerical information we mean information presented as numbers, tables or graphs.

 1  2  3  4  5  6

Never Very Often

B1. Do you own a cell phone?

 Yes

 No

B2. Do you currently have a Smart phone such as an iPhone, Android, or Windows phone?

 Yes

 No

B3. Do you ever go on-line to access the Internet or World Wide Web, or to send and receive e-mail?

 Yes

 No

C1. Is there a place that you usually go to when you are sick or need advice about your health?

 Yes

 There is no place I usually go

 There is more than one place

C2. What kind of place do you go most often?

 Clinic or health center

 Doctor’s office

 A hospital emergency room

 A hospital outpatient department

 Some other place

 Doesn’t go to one place most often

C3. Was there a time in the past 12 months when you needed medical care, but could not get it?

 Yes

 No

D1. Are you male or female?

 Male

 Female

 Other Specify: _________________________

D2. What is your age?

 _________Years

D3. What is your current occupational status?

*Mark only one.*

 Employed – fulltime

 Employed – part time

 Unemployed

 Homemaker

 Student

 Retired

 Disabled

 Other-Specify ________________________

D4. What is the highest grade or level of schooling you completed?

 Less than 8 years

 Eight through 11 years

 12 years or completed high school

 Post high school training other than college (vocational or technical)

 Some college

 College graduate

 Postgraduate

D5. Do you have any kind of health care coverage, including health insurance or government plans?

 Yes

 No

D6. Which one of these phrases comes closest to your own feelings about your household’s income these days?

 Living comfortably on present income

 Getting by on present income

 Finding it difficult on present income

 Finding it very difficult on present income


